# Round the Hospitals

**Published:** 1917-04-14

**Authors:** 


### ROUND THE HOSPITALS.

Objection was taken in Ireland to the inclusion of the word "British" in the title of the Royal College of British Nursing. It was claimed by the President of the Royal College of Physicians, Ireland, Dr. O'Carroll, that Ireland's identity was lost in such a title. Irishmen knew better than the President, and "Shamus," in an interesting article in the *Weekly Irish Times*, pointed out, with great respect to the Chairman, that the word "British" includes Ireland. "The United Kingdom of Great Britain and Ireland is otherwise known as the British Isles. The Royal British College of Nursing would obviously be as incomplete without the Irish branch of the profession as the British Isles would be if Ireland were effaced from the map. The British flag includes the Cross of St. Patrick as well as the crosses of St. George and St. Andrew. We never think of British troops or the British Navy as purely English, or English and Scottish, but as the Navy and Army which belong to the British Isles. Tnis is not a modern idea, nor an English imposition. It is as old as history. In the 'De Mundo' of Aristotle (about 350 B.C.) occurs the passage: 'Beyond the Pillars of Hercules (Straits of Gibraltar), the ocean flows round the earth, and in it are two very large islands called British (Bretanikai Legomenai), Albion and Ierene, lying beyond the Keltoi.'" "Shamus" ends with the query, "How many Irish Doctors are members of the British Medical Association, and how many Irish men and women are proud to be enrolled as British Red Cross Workers? . . . The Ancient Britons inhabited the whole territory of the British Isles."

Our readers, and the majority of the nursing profession at any rate, commenced their Easter with the belief that Mr. Paterson's letter and the two he enclosed had exploded "the Secret Compact" by revealing the ridiculous mouse. After the Easter holiday they discovered, however, that once again the Lady's fascination for treating British nurses as a body as noodles and know-nothings has yielded a miraculous result, and the mountain has produced again. The Journal devotes less than two columns to Mr. Paterson and his enclosures, but fills two and a quarter columns more with the usual "few words of criticism." Those "few words" include the following elegant periods: "mysterious pact"; "specious platitudes"; "the deprivation of the Nursing Profession of its Royal Charter" (according to history the Charter was deserted in a huff and hurry, for the Lady ran away, but the British nurses remained, with their Charter, in possession); "Economic Servitude for Trained Nurses, Throughout the Kingdom"; "bureaucratic impertinence"; "Mr. Stanley and Sir Cooper Perry to Govern the Nursing Profession by Divine Right"; "feudalistic despotism"; "let us thank God for her" (the Lady who, "not to be controlled like a fool, has secured publicity"); "egregious demands" (which, those of the Lady or of the College?); the Royal Charter as "the Feudal Phœnix to Arise from The Ashes of Our Old Liberties"; "this little Oligarchy of Men," out "to Subjugate The Whole Nursing Profession" and to "Break it on The Wheel of Despotism." Finally, the mountain points to those "Matrons on The College Council" who, wise or base women as you choose, have excluded "the rank and file from direct representation" on the Council?? We have quoted a fraction only of the "few words of criticism," which might have been made in Germany; the choicest amongst them are mere leaves of verbiage used to bury the truth out of sight of British Nurses, who will here find themselves treated as Mere Babes in the Wood. Further, the conviction of the Lady that British Nurses are in fact Noodles and Know-nothings was carried to the Danger Zone by inviting them to Swallow the Mountain and the Mouse to oblige her, because she suggests it in Prodigious Words. We have had nearly fifty years' association with British nurses, who are quite capable of taking care of themselves. They have no intention whatever to allow themselves to be carried away on the train of The Lady, to a destination and end which do not attract them, and might deprive British nurses of the Royal Charter and Royal College, both of which they have at present practically in possession.

Dr. Bygott, the medical officer of health for West Suffolk, speaking at the annual meeting of the Suffolk Nursing Association at Bury St. Edmunds, stated that his experience, when on relief duty in first-class London hospitals, was that the ignorance prevailing amongst hospital secretaries and ward sisters with regard to district nurses and their work was lamentable. District nurses were invaluable to hospitals by reason of the indirect help they give by attending to cases which would otherwise have to remain longer in the hospitals. Dr. Bygott further expressed the opinion that after the war, in consequence of the enormous slaughter of men, and the number of marriages with unsuitable women now rushed into by young men in the Army, who under other circumstances would have waited for a better type of woman before getting married, there would be a splendid type of young women in considerable numbers set free to serve not only as district nurses, but as elementary school teachers, who were at present greatly in demand. Women of refinement were required for these positions, and they would have splendid opportunities to do valuable work for the Empire. Dr. Bygott expressed a wish that a better type of women may be found to train as midwives, for he knew as an inspector of midwives under the County Council that there were still unsatisfactory women employed in this capacity.

After the war, when the Empire is knit more closely together than ever before, lessons will no doubt be learnt from the administration of the hospitals and nursing systems in Australia and New Zealand. The Annual Conference of Hospital Boards at Wellington this year, with a view to

**ROUND THE HOSPITALS—(continued).**

obtain uniformity in administration and practice, passed a resolution to the effect that it is now opportune for the adoption of a classification scheme and a uniform scale of salaries to be paid to the nursing staffs of the various hospitals and Charitable Aid Boards of the Dominion. The Conference referred the matter to the Inspector-General with a view to its taking concrete shape. A wish was expressed that the regulations under the Nurse Registration Act should be amended in order that the matron or superintendent of nurses may not be made the sole arbiter in dismissing pupil-nurses of three months' training without reference to the Hospital Board, and to abolish the rule which at present makes it necessary for the Registrar of Nurses to consent to the termination of the training of pupil-nurses.

---

Hospital Boards in New Zealand show much practical interest in the nursing staff. The Annual Conference this year recommended the Government to amend the Pensions Act to provide that where old-age pensions are misused so as to become forfeited the stipendiary magistrate shall have power to make the sum payable to the Hospital and Charitable Aid Board of the district in which the pensioner resides, such Board to be authorised to allot in goods or kind the amount of the pension by weekly grants. This is a very merciful and liberal proposal, which should be freed from many obvious dangers by placing the control and action in these cases in the hands of the Hospital and Charitable Aid Board of the district.

---

Lambeth Guardians record that a serious shortage is occurring in the number of applicants for the staff of probationer-nurses. Very few suitable replies were received from the last advertisements. This is probably due in part to the fact that several other infirmaries were advertising at the same time and offering a higher rate of pay—*e.g.*, Bermondsey Parish, £16 per annum, rising to £24 per annum; St. Pancras Parish, £12 per annum, rising to £22 per annum. The matron suggested that the Guardians should grant a war bonus, and the Board adopted a recommendation that it is desirable that the remuneration should be increased temporarily, and voted a war bonus of £5 a year.

---

On Wednesday, April 18, at 8 p.m., a meeting on behalf of the Royal College of British Nursing is to be held at Queen Mary's Hospital, Stratford. The Hon. Arthur Stanley, C.B., M.P., will address the meeting, and it is hoped that the nursing staff not only of Queen Mary's Hospital, but of all other hospitals and institutions in the neighbourhood, will attend and take part in the proceedings.

For Matrons' and Sisters' Appointments, see p. iv.

